# Quality of Life in Patients with Psoriasis Seen in the Department of Dermatology, Antananarivo, Madagascar

**DOI:** 10.1155/2020/9292163

**Published:** 2020-09-14

**Authors:** Fandresena Arilala Sendrasoa, Naina Harinjara Razanakoto, Volatantely Ratovonjanahary, Onivola Raharolahy, Irina Mamisoa Ranaivo, Malalaniaina Andrianarison, Mendrika Fifaliana Rakotoarisaona, Niriantsoa Avotriniaina Rakotonaivo, Moril Sata, Lala Soavina Ramarozatovo, Rabenja Fahafahantsoa Rapelanoro

**Affiliations:** Department of Dermatology, Faculty of Medicine, Antananarivo, Madagascar

## Abstract

**Background:**

Psoriasis is a chronic, inflammatory, and multifactorial dermatosis that impairs quality of life (QoL). Health-related QoL has become an important element in medical decision-making along with the effectiveness and the harmlessness of the treatments.

**Objective:**

To assess the impact of psoriasis in the QoL of patients with psoriasis by using the DLQI scales.

**Methods:**

A cross-sectional study from January to June 2018 was conducted in the Department of Dermatology of the University Hospital Joseph Raseta Befelatanana, Antananarivo, Madagascar, including patients more than 18 years old with mild to severe psoriasis. The severity of psoriasis was assessed using the “Psoriasis Area and Severity Index (PASI)”. QoL of patients with psoriasis was evaluated by using the DLQI scales.

**Results:**

80 patients were included, their mean age was 36.5 years, and the male to female was 1.5 : 1. The mean DLQI score was 13.8. Symptoms, feelings, and psychic were the most altered dimensions. QoL was impaired in young patients, single, having medium level education. Even though patients with disease duration more than 5 years had higher DLQI score than other patients, the difference was not statistically significant (*p* = 0.36). Furthermore, the clinical presentation of psoriasis did not influence the patient's QoL (*p* = 0.73). Patients with nail involvement had QoL impaired but the difference with another localization was not statistically significant (*p* = 0.2). The quality of life was influenced by body area involved. The higher the body surface area involved, the more QoL is impaired (*p* = 0.002). Furthermore, the higher the PASI, the more QoL is altered (*p* = 0.002).

**Conclusion:**

Psoriasis has a negative impact in the quality of life in Malagasy patients with psoriasis, especially in younger and single patients. Worse quality of life is correlated to severity of psoriasis.

## 1. Background

Psoriasis is an immune-mediated, chronic, inflammatory skin disease which affects 0.9 to 8.5% of the world's adult population [1]. This prevalence was 2% in the adult population according to data in 2011 in the unique reference center of dermatology in Madagascar [2]. It is a complex, multifaceted skin disease that may have a major impact on the patient's quality of life, influencing daily, social activities, and all other aspects of life [3].

The diagnostic is essentially clinical. It is characterized by well-delimited erythematous-desquamative plaques, which evolve with flare-ups. Disease severity is variable: mild, moderate, and serious forms. The severity of psoriasis is measured in terms of the appearance of the lesions and body surface involved; however, the degree of clinical involvement is not indefectibly correlated with the degree of physical impairment perceived by the patient [4].

The treatments control the evolution of the disease, allowing more or less complete transient regression of the lesions, which is adapted to the severity of disease and the impact on the patient's quality of life.

Depending on the severity and localization of psoriasis, patients may suffer from physical discomfort and significant disability. They may be embarrassed by their appearance and have low self-esteem due to social rejection or psychological difficulties. Many studies have described the various ways in which psoriasis can affect a patient's life [5, 6]. However, no previous data about the impact of psoriasis on the quality of life (QoL) of Malagasy patients was reported. So, this study aims at describing the extent to which psoriasis affects the QoL of patients seen in the Department of Dermatology of the University Hospital Joseph Raseta Befelatanana, Antananarivo, Madagascar.

## 2. Methodology

A cross-sectional study from January to June 2018 was conducted in the Department of Dermatology of the University Hospital Joseph Raseta Befelatanana, Antananarivo, Madagascar, which is the only reference center on Dermatology in Madagascar. Patients more than 18 years old with mild to severe psoriasis, seen in the Department of Dermatology of the University Hospital Joseph Raseta Befelatanana, Antananarivo, were included. Patients were excluded if they had a significant comorbid condition likely to impact QoL exclusive of psoriasis. We informed them about the purpose of this study, and written informed consent was obtained.

Demographic data and medical history were collected by a trained research assistant. Patients had physical examination on their date of selection. The patients had a definite diagnosis of psoriasis confirmed by clinical assessment. The dermatologists assessed the severity of their eruptions using the “Psoriasis Area and Severity Index (PASI)” at the same time. The PASI is the most tools used to quantify disease severity in psoriasis taking into account erythema, infiltration, scale, and area involved. The scores are calculated by simple addition based on the extent of the symptoms. A single score between 0 = no psoriasis and 72 = very severe psoriasis is obtained, with PASI being interpreted as >12 = severe, 7 − 12 = moderate, and <7 = mild [7].

The patients were asked to answer the questions listed in the “Dermatology life quality index (DLQI)”. The DLQI, which is a dermatology-specific tool to measure health-related quality of life, consists of ten item including 6 dimensions (symptoms and feelings, daily activities, leisure, work and school, personal relationships, and treatment), with four possible responses to each item: 0 (not at all), 1 (a little), 2 (a lot), and 3 (very much). Responses are calculated for the total DLQI score, which is 0-30 [8]. The higher the score is, the lower the quality of life. DLQI scores from 0-1 mean no effect of the disease on the patient's quality of life, scores of 2-5 mean a small effect, scores of 6-10 mean a moderate effect, scores of 11-20 correspond to a great effect, and scores of 21-30 mean a very important effect of disease on the patient's quality of life [9].

## 3. Statistical Analysis

Statistical analysis was performed using Epi info 7 software.

Student's test, chi-square, and Fisher's exact test were used for quantitative and qualitative variables, respectively. *p* ≤ 0.05 is considered statistically significant.

## 4. Results

### 4.1. Clinical Features of the Study Population

Out of 92 patients with psoriasis seen during the period of our study, 3 were excluded (due to comorbidities: diabetes and stroke) and 9 declined participation. 80 patients who accepted to answer the questionnaire were included in this study. The mean age was 36.5 years, and 60% were men (sex ratio: 1.5). Most patients were married (54%) and had received a medium-length education (51%). Among all patients, 64% were employed and 28% were students. 50% of patients presented psoriasis less than 1year and 33% between 1 and 5 years. There were 64% patients with psoriasis vulgaris, 22% with guttate psoriasis, 9 patients with erythrodermic psoriasis, and 2 patients with pustular psoriasis. 42 patients had scalp involvement.

Less than 30% of the body surface area (BSA) was involved in 36 patients (46%) and more than 30% in 44 patients (54%). The mean PASI score was 9.2. Forty-eight patients (60%) had the PASI score < 7; 22 patients (27%) with the PASI score from 8 to 12 and 10 patients presented severe form of psoriasis with the PASI score > 12.

### 4.2. Evaluation by the DLQI Score

The mean DLQI score was 13.8. Symptoms, feelings, and psychic were the most altered dimensions. Sexual dimension was altered only in 2.5% of patients. A score higher than 10 indicates that the patient's life is being severely affected by their skin disease. Fifty percent of patients reported a great effect of psoriasis in their QoL (DLQI: 11-20), 2% had a very important effect (DLQI>21), 35% had a moderate effect (DLQI: 6-10), 11% had a small effect (DLQI: 2-5), and only 1% had no effect of the disease on his quality of life.

### 4.3. Correlation between the Demographic Data and DLQI Score

Twenty-nine patients (63%) ≤37 years old had DLQI > 10 compared with 17 patients (37%) >37 years old with DLQI ≤ 10 (*X*^2^ 4.82; *p* = 0.015).

Sex and quality of life were found to be unrelated (*X*^2^ 0.67; *p* = 0.21).

Compared to another marital status, QoL was more impaired in single patients than was associated with the quality of life in patients with psoriasis (Fisher's exact test: *p* = 0.008). Furthermore, compared to employees, students had a more impaired quality of life (Fisher's exact test: *p* = 0.002, OR: 0.17, CI 95% [0.04; 0.61]). Patients with medium level education had quality of life more impaired than other groups (Fisher's exact test: *p* = 0.03).

### 4.4. The Clinical Presentation of Psoriasis and QoL

Even though patients with disease duration more than 5 years had higher DLQI score than other patients, the difference was not statistically significant (*p* = 0.36). Furthermore, the clinical presentation of psoriasis did not influence patient's QoL (*p* = 0.73).

Even though patients with nail involvement had quality of life impaired, the difference with another localization was not statistically significant (*p* = 0.2).

However, the quality of life was influenced by the body surface area involved. The higher the body surface area involved, the more quality of life is impaired (Fisher's exact test: *p* = 0.006, OR: 3.76, CI 95% [1.37; 10.85]). Distribution of participants via the DLQI score according to the sociodemographic and clinical data was shown in Table 1. Furthermore, the higher the PASI, the more QoL is altered (Student's test: *p* = 0.002). The correlation between the severity of psoriasis and QoL was shown in Table 2. The impact on QoL was classified in two groups: DLQI ≤ 10 and DLQI > 10. The skin disease is having a very large effect on the patient's life if the DLQI score is greater than 10.

## 5. Discussion

Our data show that there are no correlation between sex, educational status, and quality of life in psoriatic patients. Younger age, marital status, extensive body surface area involved, and high PASI were significantly correlated with a poor QoL. These results may raise some discussion.

The mean DLQI score in our study was 13.8. DLQI > 10 indicating significant impairment of QoL was found in 42 patients (52.5%). The symptom and psychic are the more altered dimensions. This score is higher than the results reported by several authors (Table 3) [10–15]. However, Maoua et al. reported a very poor QoL in psoriatic Tunisian patients (mean DLQI: 16.1); symptoms and psychic were the dimensions most altered [16].

In our study, younger age was statistically significant associated with poor QoL (*p* = 0.015). It is possible that young people assess their quality of life better than older people, and the disease duration is not yet long. It is consistent with other findings reported by Amy de la Breteuque et al. (correlation statistically significant between age and QoL, *p* < 0.006) [17] and Kelati et al. who used skindex16 to evaluate the QoL in psoriatic patients [18]. Young people have more professional responsibilities and interpersonal relationship and therefore have difficulty accepting their illness.

Gender and quality of life were found to be unrelated in our study. It is consistent with the result reported by Kouris et al. [15]. Generally, there are no difference in the severity of physical symptoms suffered by men and women. Anterior controversial results have been reported by several authors regarding the correlation between gender and quality of life in psoriasis. Most of studies reported that female had poor quality of life compared to male with psoriasis [19–21]. However, Valenzuela et al. in Chilean patients reported that men were consistently more affected in almost all areas [13], which is rarely reported before.

Marital status was a determinant of quality of life in psoriasis patients in our study (correlation *p* = 0.008). Single patient had poor quality of life. It can be explained by the fact that single people take more care of their physical appearance and have trouble accepting their chronic illness.

Even though patients with disease duration more than 5 years had higher DLQI score than other patients, the difference was not statistically significant (*p* = 0.36). Kelati et al. showed that the disease duration impaired the QOL in psoriasis (*p* = 0.009), mainly on the emotion dimension, depression, and frustration (*p* = 0.007) [18]. However, Valenzuela et al. showed that patients with recent psoriasis (<5 years) had a more impaired QoL than those with psoriasis over 20 years (*p* = 0.00007) [13]. The difference between several studies may result from the cultural, social, and economic differences of the groups studied.

The localization of psoriasis is a determinant factor of QoL in several studies. In our study, even though the difference between localizations was not statistically significant (*p* = 0.2), patients with nail involvement had quality of life impaired. Lin et al. reported also that nail involvement was associated significantly with QoL altered [22]. Benchikhi et al. and Yang et al. reported that DLQI high scores in psoriasis were significantly associated with uncovered areas involvement particularly in the personal relationship area [14, 23].

Our study confirmed the influence of the body surface area involved with QoL, and higher BSA involved was positively correlated with QoL impaired (*p* = 0.002). Our result was consistent with those reported by several authors [14, 18, 22]. Strober et al. reported that DLQI worsened with disease severity evaluated by the BSA (affected body surface area) score and IGA 2011 scale (Investigator's Global Assessment) [24].

Most quality of life scales are validated in correlation with the severity of the disease. So, the doctor's point of view meets the patient's point of view. However, several previous data indicate variable results. High PASI was associated to poor QoL in our study (*p* = 0.002). It is consistent with results reported by several authors [15–17]. A multicentre, prospective study conducted in Spain found psoriasis severity was the primary factor affecting QoL, using PASI for the multivariate modeling [25]. However, other results reported by Benchikhi H, Yang HJ, and Amy De la Breteuque et al. showed a negative correlation between PASI and QoL [13, 17, 23]. This discrepancy between the scales of QoL and PASI shows that the management of a psoriatic patient should include both physical and psychological dimensions.

This study was a cross-sectional analysis, which does not allow for causal interferences regarding psoriasis severity and the outcomes of interest. Furthermore, the subjectivity of the quality of life responses may interfere with the results of this study.

Even though our result is not the representative of the general population due to the small sample size (participants at a single institution and the duration of the study is short), it will contribute to understanding the impact of psoriasis in patient's quality of life. Our department is the unique dermatology center in Madagascar where 6 dermatologists work, and there is no dermatologist in private practice.

## 6. Conclusion

Younger age, marital status as single, medium level education, and increased psoriasis severity were the determinant factors of QoL in patients with psoriasis seen in the Department of Dermatology, Antananarivo, Madagascar. No correlation was found between gender, localization of psoriasis, and QoL. Better understanding and communication between psoriasis patients and their physicians may help to improve not only the clinical outcomes in psoriaris but also the patient's QoL.

## Figures and Tables

**Table 1:** Distribution of participants via the DLQI score according to the sociodemographic and clinical data.

	Patients with DLQI≤10 *n* (%)	Patients with DLQI>10 *n* (%)	*p*
Age			
≤37 years (*n* = 45)	17 (37)	29 (63)	0.015
>37 years (*n* = 35)	21 (62)	13 (38)	
Gender			
Male (*n* = 48)	21 (44)	27 (56)	0.21
Female (*n* = 32)	17 (53)	15 (47)	
Educational status			
Elementary school (*n* = 15)	7 (47)	8 (53)	
Secondary school (*n* = 41)	8 (20)	33 (80)	0.03
University (*n* = 24)	11 (46)	13 (54)	
Marital status			
Single (*n* = 33)	9 (27)	24 (73)	
Married (*n* = 43)	25 (58)	18 (42)	0.008
Divorced (*n* = 1)	1 (100)	0 (0)	
Widower (*n* = 3)	3 (100)	0 (0)	
Occupation			
Students (*n* = 22)	4 (18)	18 (82)	0.002
Employees (*n* = 58)	33 (57)	25 (43)	
Duration of disease (years)			
≤ 1 (*n* = 40)	22 (55)	18 (45)	
[1-5] (*n* = 26)	12 (46)	14 (54)	0.36
[5-10] (*n* = 5)	1 (20)	4 (80)	
> 10 (*n* = 9)	3 (33)	6 (67)	
Clinical presentation			
Psoriasis vulgaris (*n* = 51)	26 (51)	25 (49)	
Guttate psoriasis (*n* = 17)	6 (6)	11 (64)	0.73
Erythrodermic psoriasis (*n* = 9)	4 (44)	5 (55)	
Pustular psoriasis (*n* = 2)	1 (50)	1 (50)	
Localization of the psoriasis			
Scalp (*n* = 42)	17 (40)	25 (60)	
Nail (*n* = 4)	1 (25)	3 (75)	0.2
Skin fold (*n* = 2)	1 (50)	1 (50)	
Palmo-plantar (*n* = 3)	3 (100)	0	
Body surface area involved			
≤ 30% (*n* = 35)	23 (66)	12 (34)	
> 30% (*n* = 45)	15 (33)	30 (67)	0.006

**Table 2:** Correlation between the severity of psoriasis and QOL.

DLQI	Number of patients	Mean of PASI	Standard deviation of PASI	*p*
≤10	38	5.23	3.3	0.002
>10	42	10.38	4.67	

**Table 3:** DLQI in psoriasis reported by several authors.

Authors/country	Year	Number of cases	Mean DLQI
Mazotti et al., Italie [7]	2005	900	8
Wah et al., Norvège [8]	2006	85	10.6
Eghileb et al., Angleterre [9]	2007	63	10
Valenzuela et al., Chile [10]	2010	153	14
Benchikhi et al., Maroc [11]	2013	40	11.15
Kouris et al., Grec [12]	2015	84	12.61
Maoua et al., Tunisie [13]	2015	58	16.1
Our study	2018	80	13.85

## Data Availability

The data used to support the findings of this study are available from the corresponding author upon request.

## Abbreviations

QoL: Quality of life
DLQI: Dermatology life quality index
PASI: Psoriasis area and severity index
BSA: Body surface area
UH/JRB: University hospital Joseph Raseta Befelatanana.
